# Association between Milk Intake and All-Cause Mortality among Chinese Adults: A Prospective Study

**DOI:** 10.3390/nu14020292

**Published:** 2022-01-11

**Authors:** Xiaona Na, Hanglian Lan, Yu Wang, Yuefeng Tan, Jian Zhang, Ai Zhao

**Affiliations:** 1 Vanke School of Public Health, Tsinghua University, Beijing 100084, China; nxn21@mails.tsinghua.edu.cn (X.N.); mindylan678@gmail.com (H.L.); tanyf1995@163.com (Y.T.); 2 Institute for healthy China, Tsinghua University, Beijing 100084, China; 3 School of Public Health, Peking University, Beijing 100091, China; ywang@bjmu.edu.cn (Y.W.); zhangjian92@pku.edu.cn (J.Z.)

**Keywords:** milk intake, all-cause mortality, dietary quality, dietary diversity, energy intake

## Abstract

Background: Little is known about the effect of milk intake on all-cause mortality among Chinese adults. The present study aimed to explore the association between milk intake and all-cause mortality in the Chinese population. Methods: Data from 1997 to 2015 of the China Health and Nutrition Survey (CHNS) were used. A total of 14,738 participants enrolled in the study. Dietary data were obtained by three day 24-h dietary recall. All-cause mortality was assessed according to information reported. The association between milk intake and all-cause mortality were explored using Cox regression and further stratified with different levels of dietary diversity score (DDS) and energy intake. Results: 11,975 (81.25%) did not consume milk, 1341 (9.10%) and 1422 (9.65%) consumed 0.1–2 portions/week and >2 portions/week, respectively. Milk consumption of 0.1–2 portions/week was related to the decreased all-cause mortality (HR: 0.59, 95% CI: 0.41–0.85). In stratified analysis, consuming 0.1–2 portions/week was associated with decreased all-cause mortality among people with high DDS and energy intake. Conclusions: Milk intake is low among Chinese adults. Consuming 0.1–2 portions of milk/week might be associated with the reduced risk of death among Chinese adults by advocating health education. Further research is required to investigate the relationships between specific dairy products and cause-specific mortality.

## 1. Introduction

Milk and dairy products play critical roles in providing multiple essential nutrients and energy for humans [1] and are listed as a core part of dietary recommendations around the world [2,3,4]. The Dietary Guideline for Americans recommended intake of fat-free and low-fat milk and dairy products is three servings/day (about 710 mL/day) for adults [2]. Chinese Dietary Guidelines in 2016 (CDG–2016) has a recommended consumption of 300 g of milk and dairy products every day to maintain adequate nutrition [3].

Although full-fat or low-fat milk has been proved to prevent multiple chronic diseases such as fractures, diabetes, and cancer, together with mortality [5,6,7,8], recent literature has offered contradictory findings of the health effects of milk. Several systematic reviews and meta-analyses have concluded that milk intake was not related to cardiovascular disease, cancer, and all-cause mortality [9,10,11]. More seriously, studies over the past decades have provided evidence that higher milk intake increased the risk of fracture, coronary heart disease, cardiovascular mortality, and all-cause mortality in developed countries [12,13,14,15]. It seems that the optimal intake of milk for individuals would depend on dietary structure, that is, milk can improve nutrition and further prevent chronic diseases and mortality among populations in developing countries but is likely to be profitless, even detrimental, for those in developed countries with high dietary quality [16].

Although milk intake has been gradually increased in China, it is still at a low level because of perennial dietary habits [17]. The average milk and dairy product intake has risen from 2.06 g/day in 1989 to 26.47 g/day in 2011 [18]. A cohort study of 45,411 individuals in China has shown that median (*P*_25_, *P*_75_) milk and its product intake in China was 20.5 (5.19, 75.0) g/day [17] during 1999 to 2010 [5], which is significantly lower compared to European and North American countries (368.4 ± 282.5 g/day) [17], and also far lower than the Chinese recommended amount (300 g/day) [19]. As a result of insufficient intake of milk and dairy products, calcium intake is insufficient among Chinese adults. One recent cohort study reported the calcium intake was 517.0 (SD: 266.4) mg/day among middle-aged Chinese, which is lower than the Estimated Average Requirements (650 mg/day) of Chinese Dietary Reference Intakes in 2013 [20].

The health effects of milk, a major dietary source of calcium, among Chinese adults may be very different from those in developed countries. In addition, in the context of unbalanced status of economic development, culture, and lifestyle in China, nutrition status and quality among Chinese adults are especially different. Therefore, the role of milk plays might be quite different with different dietary diversity and energy intake. Nevertheless, little Chinese research has revealed the health effects of milk on certain diseases [21,22,23], and little is known about the effect of milk intake on all-cause mortality among Chinese adults, especially for Chinese with different dietary quality.

To address these gaps in the previous research literature, the present study aimed (1) to explore the association between milk intake and all-cause mortality, and (2) to further explore the association among people with varied dietary quality.

## 2. Materials and Methods

### 2.1. Study Population

Data used in this study were obtained from China Health and Nutrition Survey (CHNS), which is a large-scale, ongoing longitudinal and household-based survey initiated in 1989; nine additional waves were collected in 1991, 1993, 1997, 2000, 2004, 2006, 2009, 2011, and 2015. The detailed study design can be found on the study website [24]. Briefly, a multistage random cluster sampling was used to select samples representing the socioeconomic status and health indicators of the population in 11 provinces (Guizhou, Heilongjiang, Henan, Hubei, Hunan, Jiangsu, Liaoning, Shaanxi, Shandong, Yunnan, and Zhejiang) and four autonomous cities/regions (Beijing, Shanghai, Chongqing, and Guangxi) of China.

**Figure 1:**
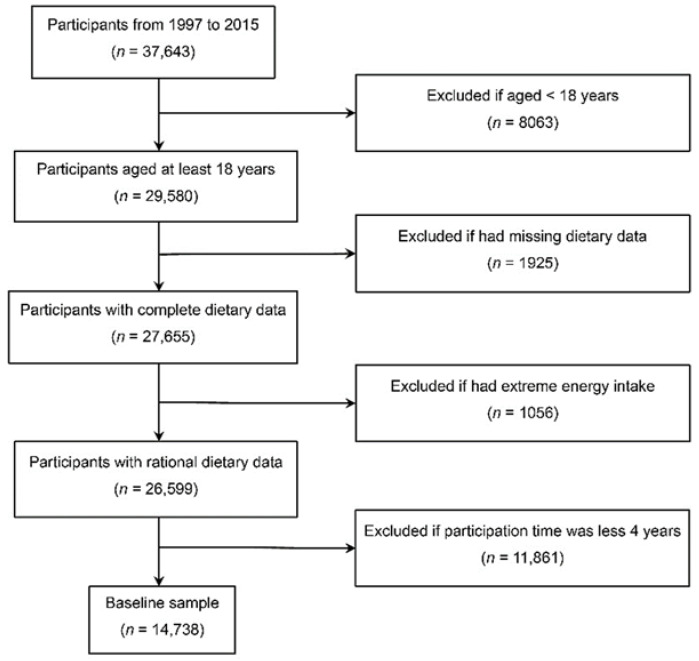
Flow chart of sample selection.

To investigate association between milk intake and all-cause mortality, our sample included data in the seven waves collected from 1997 to 2015 (dietary data in 2015 were not available). Data in 1989 and 1993 was excluded because dietary data in the two years were not correctly coded and could not be identified. Participants were excluded with: (1) baseline age <18 years or missing; (2) missing dietary data; (3) extreme average energy intake with <520 kcal/day or >8000 kcal/day in all the waves of participation; or (4) participation time <4 years. A total of 14,738 participants were included in this study (Figure 1).

### 2.2. Data Collection

Dietary data were collected by trained interviewers over three consecutive days within a week at individual and household levels. The three consecutive days were selected randomly from Monday to Sunday. For individual dietary intake, all the foods consumed (meals and snacks) by participants over the previous 24 hours were reported. Types, quantities of all food consumed, and dining places were recorded by the interviewers with the help of food models and pictures. Household food and condiment consumption were calculated by recording the changes of inventory from the beginning to the end of a three-day survey, including all purchased, homemade, and processed food [25].

Milk was identified as liquid milk, yogurt, and milk powder (100 g yogurt or 12.5 g milk powder were converted to 100 g liquid milk according to their protein ratios in fresh milk according to CDG–2016) [3]. Other types of dairy products were not included because of their rare intake in the studied population. The cumulative average intake of milk was calculated by summing milk quantity and dividing by the number of waves from participating in the survey to death or loss to follow-up during 1997–2015. Weekly consumption of milk was divided into portions: one portion of milk was regarded as 300 g according to CDG–2016 [3]. Milk consumption was categorized into three groups as follows: no consumption, 0.1–2 portions/week, and >2 portions/week. Cumulative average intakes of vegetables, fruits, and red meat during follow-up, and milk intake at the entry to the cohort were calculated.

Dietary quality was evaluated in this study by dietary diversity score (DDS) and energy intake. DDS was developed according to CDG–2016 [3], which contains 10 food groups, eight of which (cereals and tubers, vegetables, fruits, meat, soybeans and nuts, eggs, aquatic products, as well as milk and dairy products) were involved in developing the DDS. Since salt and oil are indispensable in the Chinese diet, they were not used to establish DDS. The DDS was classified as low or high according to its median. The nutrient intake and daily energy intake were calculated based on the Chinese Food Composition Tables [26,27]. Similarly, daily energy intake was classified as low or high according to the median intake level.

### 2.3. Assessment of Outcome

All-cause mortality was assessed according to information reported in each wave of the survey, with updating of live or deceased status in the household registration system. Dates of death were recorded if a decease occurred. Years of follow-up were calculated from the enrolment time (1997) to death or censoring.

### 2.4. Definition of Covariates

Participants were required to complete a questionnaire and a physical examination with the assistance of trained surveyors in each wave. The questionnaire contained: (1) sociodemographic information, including age (continuous variable), sex (male or female), education (junior high school or below, senior high school or vocational school, university or above, and unknown), place of residence (eastern, central, and western China), and individual income (<30,000 yuan/year, 30,000–59,999 yuan/year, and ≥60,000 yuan/year); (2) information about personal lifestyle behaviors, including smoking status (never, former smoker, current smoker, and unknown), and alcohol intake (never, <1 time/week, ≥1 time/week, and unknown), physical activity (MET-hour/week); and (3) information about chronic disease history (no or yes), including hypertension, diabetes, fracture, and cancers. Height and weight were measured by surveyors with standard methods. Body mass index (BMI) was calculated by dividing weight (kg) by the square of height (m^2^) and divided into four levels according to the Working Group on Obesity in China: <18.5 kg/m^2^, 18.5–23.9 kg/m^2^, 24–27.9 kg/m^2^, and ≥28 kg/m^2^ [28]. The above information might slightly change with time, so sociodemographic characteristics, lifestyle behaviors, and BMI at baseline were collected, and an occurrence of chronic disease in any wave was identified as having a chronic disease history in this study. 

### 2.5. Statistical Analyses

Chi-square (*χ*^2^) tests and one–way analyses of variance (ANOVAs) were used to compare basic categorical and continuous variables of the milk intake groups. The association between milk and all-cause mortality was explored using Cox proportion hazard regression and further stratified with different levels of DDS and energy intake. The Cox proportion hazard regression included two adjusted models: Model 1 adjusted for age, sex, education, place of residence, and individual annual income; Model 2, based on Model 1, further adjusted for smoking status, alcohol intake, physical activity, BMI, chronic disease history, vegetable intake, fruit intake, red meat intake, DDS, and energy intake. The same procedure mentioned above was conducted for stratified analyses of dietary quality (including DDS and energy intake). DDS and energy intake were not adjusted in their corresponding stratified analyses. Restricted cubic spline (RCS) Cox regression was additionally performed to explore the nonlinear dose–response relationship between milk intake and all-cause mortality, adjusting for the same confounders as Model 2 in Cox proportion hazard regression. The number of knots with max *R*^2^ and *Dxy* in the RCS Cox regression model was selected. In sensitivity analysis, we applied inverse probability of treatment weight (IPTW) Cox proportion hazard regression to balance off the confounding factors between milk intake and all-cause mortality. In short, as a type of propensity score method, IPTW balances the distributions of confounding factors among different milk intake groups in the analysis phase, and its strength is no loss of sample size compared to propensity score match [29]. Confounding factors considered in this study were age, sex, education, place of residence, individual annual income, smoking status, alcohol intake, physical activity, BMI, chronic disease history, vegetable intake, fruit intake, red meat intake, and milk intake at the baseline. Probabilities of participants consuming milk were calculated by multiple logistic regression. Weightings of participants in the milk consumption group were obtained by calculating the reciprocal of probabilities, and weighted Cox proportion hazard regression was applied to examine the association between milk intake and all-cause mortality. In addition, the association between milk intake at baseline and all-cause mortality was also explored.

Statistical significance was determined as *p* < 0.05 (two-tailed). Statistical analyses were performed with R 3.6.2 (R Development Core Team, Vienna, Austria).

## 3. Results

### 3.1. Sociodemographic Characteristics of Participants

Sociodemographic and dietary characteristics at baseline are presented in Table 1. 11,975 (81.25%) did not consume milk (no consumption), 1341 (9.10%) and 1422 (9.65%) consumed 0.1–2 portions/week and >2 portions/week, respectively. The median (*P*_25_, *P*_75_) DDS and daily energy intake were 3.27 (2.67, 4.00) and 2046.15 (1714.44, 2384.04) kcal/day, respectively. The differences in age, education, place of residence, individual annual income, smoking status, alcohol intake, BMI, chronic disease history, physical activity were significant, and sex was not statistically different among them.

### 3.2. Dietary Intake Characteristics of Participants

Dietary quality and intake of other foods are shown in Table 2. DDS, daily energy intake, consumption of vegetables, fruits, and red meat, and baseline milk intake among the three milk intake groups were statistically significant. Compared to no milk consumption, higher DDS and intake of fruits and red meat but lower intake of energy and vegetables among participants consuming milk were observed.

### 3.3. Association between Milk Intake and All-Cause Mortality

#### 3.3.1. Overall Population

Association between milk intake and all-cause mortality are shown in Table 3. Overall, during a median (*P*_25_, *P*_75_) follow-up of 10.31 (5.00, 17.84) years with 157,528.10 person-years, 642 deaths occurred. All-cause mortality was 4.30/1000 person-years for no milk consumption, 2.53/1000 person-years for 0.1–2 portions milk/week, and 3.35/1000 person-years in the group consuming >2 portions milk/week, respectively. In the unadjusted and Model 2 Cox proportion hazard regression models, consuming 0.1–2 portions/week was associated with lower all-cause mortality compared to the no milk consumption group, while no association was observed between consuming >2 portions/week and all-cause mortality.

The results of the IPTW Cox proportion hazard regression were similar to those of the aforesaid Cox regression: consuming 0.1–2 portions/week but not >2 portions/week was related to the lower risk of death in comparison to no milk consumption.

#### 3.3.2. Stratified Analyses

In the low DDS group, the results of univariate, multivariate, and IPTW Cox proportion hazard regression were similar: consumption of 0.1–2 portions/week and >2 portions/week did not change all-cause mortality compared to no milk consumption. Among participants with high DDS, in adjusted models, consuming 0.1–2 portions/week was associated with the reduction of all-cause mortality, while the phenomenon was not observed in the unadjusted or IPTW models.

In the low energy intake group, the results of all Cox proportion hazard regression showed no association of consuming milk at 0.1–2 portions/week and >2 portions/week with the risk of death compared to no milk consumption. For those with high energy intake, the unadjusted, adjusted, and IPTW Cox proportion hazard regression models showed significant associations between milk consumption of 0.1–2 portions/week and all-cause mortality.

### 3.4. Nonlinear Relationship between Milk Intake and All-Cause Mortality

**Figure 2:**
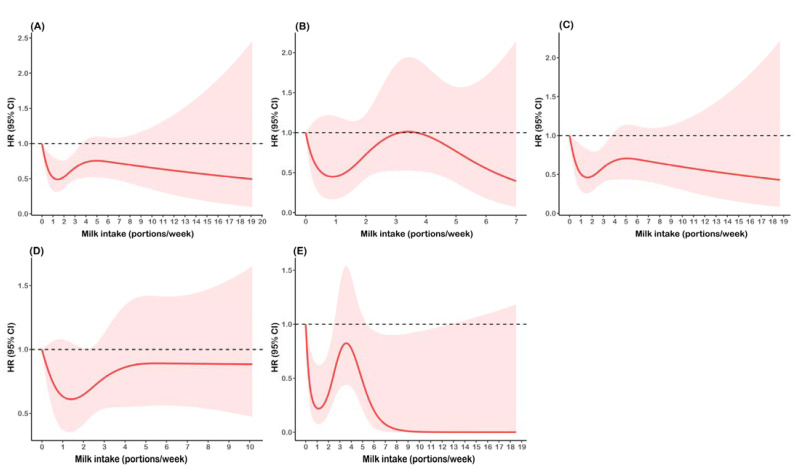
Restricted cubic spline plots to evaluate relationships between milk intake and all-cause mortality among (**A**) Overall participants; (**B**) Participants with low dietary diversity score; (**C**) Participants with high dietary diversity; (**D**) Participants with low energy intake; and (**E**) Participants with high energy intake. HR and 95% CI were adjusted for age, sex, education, place of residence, individual annual income, smoking status, alcohol intake, physical activity, BMI, chronic disease history, vegetable intake, fruit intake, red meat intake, dietary diversity score, and energy intake. Dietary diversity score and energy intake were not adjusted in their corresponding stratified analyses. Units of milk intake: portions/week (1 portion = 300 g).

Figure 2 shows the nonlinear relationships between milk intake and all-cause mortality among the overall population and subgroups stratified by dietary quality. RCS models with four knots were selected because of max *R*^2^ and *Dxy* in the models. Among the overall population, milk intake of about 0.1–3 portions/week was associated with decreased all-cause mortality, whereas higher-level intake (>3 portions/week) was not. In the stratified analyses, the association in the high DDS group were similar to the overall population, and milk intake of 0.1–2 portions was related to the lower risk of death among participants with high energy intake; however, the associations in the low DDS and low energy intake groups were not observed.

### 3.5. Sensitivity Analyses

Apart from IPTW weighted Cox proportion hazard regression, other sensitivity analyses did not substantially modify our findings. The results of the association between milk intake at baseline and all-cause mortality remained stable (Table S1).

## 4. Discussion

In this large, national, and prospective cohort study with a long follow-up time, we first explored the association between cumulative average intake and milk intake on all-cause mortality in Chinese adults. In contrast to most reports from European and North American countries, properly increased intake of milk (0.1–2 portions/week, 1 portion = 300 g) could be associated with reduction of all-cause mortality in China.

### 4.1. Insufficient Intake of Milk among Chinese Adults

As a type of nutrient-dense food, milk is rich in energy, protein, essential minerals, and vitamins, such as phosphorus, potassium, vitamins A, D, and B12, riboflavin, niacin, and especially calcium [30]. Most dietary calcium requirements cannot be met if a person does not consume milk, and it is difficult to replace nutrients from milk with nonmilk alternatives [31]. Meanwhile, milk might reduce appetite and further reduce the consumption of other foods rich in fat [32]. Therefore, milk intake might not increase the total energy intake and body weight [33,34], which is similar to our results in Table 2. Previous studies have shown that protein and calcium in milk played essential roles in preventing osteoporosis [35,36]. Furthermore, minerals in milk, such as calcium, potassium, and magnesium, were proved to contribute to maintaining healthy blood pressure and improving in vivo insulin sensitivity and insulin response [37]. However, our results showed that milk consumption among Chinese adults was obviously inadequate; most participants did not consume milk during the follow-up. Similar results have been shown by other studies [17,19]. A national review revealed that only 23.7% of participants consumed dairy every day, and the average Chinese milk intakes in large–, small–, and medium–sized cities and rural areas in 2016 were 64.3, 24.2, 9.1, and 4.9 g/day, respectively [19], which were far lower than the recommendation of CDG–2016 (300 g/day) and consumption in other countries. For example, average intakes of milk in the Netherlands and the UK were 466 g/day and 0.2 L/day (about 200 g/day), respectively [14,38]. Therefore, under the circumstances, the health effects of milk among Chinese need to be evaluated.

### 4.2. Role of Milk in Health

In recent decades, the role of milk in health has been controversial. Ming Ding and colleagues drew the conclusion from three large cohort studies in the United States that high total dairy consumption was not associated with the risk of mortality [39]. In addition, concerns exist about whether dairy/milk would result in possible adverse health outcomes [16]. Several cohort studies showed that higher dairy/milk intake increased the risk of hip fracture, cardiovascular mortality, and all-cause mortality [11,13,14,15,40].

Several recent meta-analyses have also concluded that drinking milk or dairy had no benefit to health, but even increased the risk of cancer and mortality [41,42,43], which might be explained by the fact that excessive intake of milk would increase circulating concentrations of insulin-like growth factor (IGF)-I [44]. However, the reported heterogeneity of these analyses was high, and the research samples and methods of these recruited studies were diverse.

In this study, we observed the association between milk intake of 0.1–2 portions/week and the decreased risk of death, and these results are supported by several previous studies conducted in China [5,22,23] and other countries in East Asia [45,46,47,48]. These studies reported that dairy/milk was beneficial to prevent diabetes and cardiovascular disease among Chinese adults. However, our results were discrepant with literature reports from developed countries, which showed that milk consumption was positively associated with all-cause mortality (HR: 1.13, 95% CI: 0.95–1.35) and cancer mortality (HR: 1.56, 95% CI: 1.17–2.08) [8]. The inconsistency could be explained by the different milk consumption levels among different countries. It should be noted that the highest milk intake group in our study (>2 portions/week) was still at a low level compared to developed countries, while the high level in most of the above studies was defined by several servings/day [39], and in an article concerning American adults *P*_80_ was even 17.8 (SD: 5.9) servings/day, or about (4218.6 ± 1398.3) mL/day. In addition, in the RCS Cox regression, we found that the decreased risk of all-cause mortality was seen only in the milk intake group of 0.1–2 portions/week rather than the milk intake group of >2 portions/week. This phenomenon might result from the rough grouping of milk intake, which impeded the evaluation of the actual effect of milk intake. The health effects of high milk intake in the Chinese population still need to be evaluated, especially for vulnerable groups such as children and elderly people.

### 4.3. Association between Milk and All-Cause Mortality for Different Dietary Qualities

As demonstrated by previous literature, the health effect of dairy/milk depends on people’s proper dietary and nutritional status. Our results further examined the associations between milk intake and all-cause mortality stratified by dietary diversity and the results showed that drinking 0.1–2 portions/week was associated with the decreased all-cause mortality only among people with high DDS. The results suggest that milk intake of 0.1–2 portions/week might generate higher health benefits among the population with insufficient calcium intake. While no significant association was found among the population consuming >2 portions/week, which might be explained by our rough grouping of milk intake because of the limited number of populations who consumed milk and excessive milk intake might not be beneficial and even do harm to human health. Considering synthetically the results of our study and other studies involving Chinese adults [5,21,49], milk intake of 0.1–3 portions/week might benefit the health of Chinese adults. However, the optimal amount of milk intake needs randomized controlled trials to further determine.

As an important component of DDS and a sign of a healthy diet, greater milk consumption was associated with higher dietary diversity and a higher intake of other healthy foods [50]. Recent research revealed that increased intake of milk and high DDS might be some of the reasons why the Japanese have the world’s longest life expectancy [51]. Higher DDS was also found to lower the risk of fracture and disability in activities, and benefit cognitive function and memory status among adults [52,53,54,55]. Therefore, people consuming more milk might achieve a higher DDS, which could explain the association between a higher milk intake and decreased mortality in the high DDS group.

When considering energy intake, milk consumption of 0.1–2 portions/week was related to the lower all-cause mortality in the high energy intake group. One cohort study showed that a moderate energy intake (2104 kcal/day) decreased all-cause mortality by 26%, compared to a low energy intake (1425 kcal/day) [56]. In our study, total energy intake among adults with no consumption, milk intake of 0.1–2 portion/week, and >2 portion/week were 2057.07, 1983.93, and 1996.93 kcal/day, respectively, which were lower than Chinese Dietary Reference Intake 2013 [20]. Our results of energy intake are similar to other studies conducted in China (2063 kcal/day) in 2012 [57], but lower than US adults (2170.2 [SD:5.6] kcal/day) in 2013–2014 [58] and Korean adults (2463.1 [SD: 16.7] kcal/day) in 2013–2015 [59]. Therefore, total energy intake among Chinese adults is still insufficient and in descending trend [57]. Protein in milk provides plenty of energy, scavenges free radicals, and participates in immunoregulation, so milk could decrease mortality among people with high energy intake. Therefore, as a symbol of a healthy diet, an increased intake of milk might promote good health based on the current dietary quality and structure among Chinese adults. Further studies that take variables such as milk, mortality, DDS, and energy intake into account, need to be undertaken.

### 4.4. Limitations

Several limitations of this study should be mentioned. First, only liquid milk, yogurt, and milk powder were included in our study, and other types of dairy products were not taken into consideration in analyses because of their low consumption among the Chinese. The obvious difference in the range of dairy products consumed between the people in developed countries and the Chinese might be one of the reasons for different conclusions between our study and other studies in developed countries. Second, milk intake was roughly categorized into three groups because of the limited number of participants who consumed 0.1–1 portion/week and excessive milk, so the results for 0.1–2 portions/week and >2 portions/week might be misestimated. Third, data on nutrient supplement intake were not available in the CHNS, which might misestimate the association between milk intake and all-cause mortality. Fourth, since cause-specific mortality was not available in CHNS, the contribution of milk intake to cause-specific mortality could not be identified and needs further investigation. Finally, potential confounding bias cannot be measured and adjusted completely, and residual confounding cannot be entirely ruled out. However, we identified the confounding factors as far as possible and meanwhile avoid excessive adjustment, and the stable results of sensitivity analyses suggest that our results are credible.

## 5. Conclusions

In conclusion, we observed milk intake in the current population to be far lower than the recommendation of CDG–2016. The study further revealed that the consumption of 0.1–2 portions/week of milk products might be associated with the reduced all-cause mortality, and the association remains when further stratified by the dietary quality and in sensitivity analyses, which demonstrate the reliability of our results. Therefore, the heath effect of a certain food may differ in varied populations. Combined with the fact that milk intake in the Chinese population was far lower than the recommendation of CDG–2016, health education, health promotion, and encouragement toward increased milk intake to 0.1–2 portions/week are urgently needed.

Further research is required to investigate the association between specific dairy products and cause-specific mortality among Chinese, and further determine the specific requirements of each kind of dairy. More studies on certain populations, such as children and the elderly, should also be taken into consideration.

## Figures and Tables

**Table 1:** Baseline sociodemographic and behavioral characteristics of participants.

		No Consumption	0.1–2 Portions/Week	>2 Portions/Week	*p*
		(*N* = 11,975)	(*N* = 1341)	(*N* = 1422)	
Age, *M* (*P*_25_, *P*_75_)		42.0 (32.0, 54.0)	44.0 (34.0, 57.0)	50.0 (36.0, 61.0)	<0.001
Sex, *n* (%)					0.123
	Male	5604 (46.8)	614 (45.8)	626 (44.0)	
	Female	6371 (53.2)	727 (54.2)	796 (56.0)	
Education, *n* (%)					<0.001
	Junior high school or below	6404 (53.5)	466 (34.8)	516 (36.3)	
	Senior high school or vocational school	1574 (13.1)	313 (23.3)	305 (21.4)	
	University or above	722 (6.03)	259 (19.3)	270 (19.0)	
	Unknown	3275 (27.3)	303 (22.6)	331 (23.3)	
Place of residence, *n* (%)					<0.001
	Eastern China	4340 (36.2)	791 (59.0)	950 (66.8)	
	Central China	4487 (37.5)	342 (25.5)	289 (20.3)	
	Western China	3148 (26.3)	208 (15.5)	183 (12.9)	
Individual annual income, yuan, *n* (%)					<0.001
	<30,000	6974 (58.2)	732 (54.6)	752 (52.9)	
	30,000–59,999	3535 (29.5)	435 (32.4)	496 (34.9)	
	≥60,000	1466 (12.2)	174 (13.0)	174 (12.2)	
Smoke status, *n* (%)					<0.001
	Never	5347 (44.7)	738 (55.0)	770 (54.1)	
	Former smoker	333 (2.78)	45 (3.36)	60 (4.22)	
	Current smoker	2070 (17.3)	205 (15.3)	206 (14.5)	
	Unknown	4225 (35.3)	353 (26.3)	386 (27.1)	
Alcohol intake, times/week, *n* (%)					<0.001
	Never	5162 (43.1)	655 (48.8)	696 (48.9)	
	<1	888 (7.42)	140 (10.4)	123 (8.65)	
	≥1	1665 (13.9)	189 (14.1)	211 (14.8)	
	Unknown	4260 (35.6)	357 (26.6)	392 (27.6)	
Physical activity, MET-hour/week, *M* (*P*_25_, *P*_75_)		125 (57.0, 199)	109 (56.0, 167)	96.1 (49.0, 158)	<0.001
BMI, kg/m^2^, *n* (%)					<0.001
	<18.5	354 (2.96)	34 (2.54)	45 (3.16)	
	18.5–23.9	8222 (68.7)	860 (64.1)	879 (61.8)	
	24.0–27.9	2482 (20.7)	323 (24.1)	376 (26.4)	
	≥28.0	917 (7.66)	124 (9.25)	122 (8.58)	
Chronic disease history, *n* (%)					<0.001
	No	10,741 (89.7)	1154 (86.1)	1153 (81.1)	
	Yes	1234 (10.3)	187 (13.9)	269 (18.9)	

**Table 2:** Dietary intake characteristics of participants.

		No Consumption	0.1–2 Portions/Week	>2 Portions/Week	*p*
		(*N* = 11,975)	(*N* = 1341)	(*N* = 1422)	
Dietary diversity score, *M* (*P*_25_, *P*_75_)		3.00 (2.61, 3.67)	4.00 (3.33, 4.67)	4.38 (3.75, 5.17)	<0.001
Energy intake, kcal/day, *M* (*P*_25_, *P*_75_)		2057.07 (1723.43, 2400.67)	1983.93 (1663.95, 2297.24)	1996.05 (1678.69, 2293.63)	<0.001
Vegetables intake, g/day, *M* (*P*_25_, *P*_75_)		208 (144, 282)	186 (127, 250)	192 (133, 261)	<0.001
Fruits intake, g/day, *M* (*P*_25_, *P*_75_)		0.00 (0.00, 100)	83.3 (0.00, 150)	100 (33.3, 168)	<0.001
Red meat intake, g/day, *M* (*P*_25_, *P*_75_)		1.33 (0.00, 20.0)	9.33 (0.00, 33.3)	7.32 (0.00, 33.3)	<0.001
Milk intake at baseline, potions/week, *n* (%)					<0.001
	0	11,975 (100)	307 (22.9)	179 (12.6)	
	0.1–2	0 (0)	933 (69.6)	243 (17.1)	
	≥3	0 (0)	101 (7.5)	1000 (70.3)	

**Table 3:** Association between average milk intake and all-cause mortality.

	No Consumption	0.1–2 Portions/Week	>2 Portions/Week
Overall population			
Incidence (no. of deaths/1000 person-years)	4.30	2.53	3.35
Unadjusted Model	1.00 (Reference)	0.63 (0.44, 0.90) *	0.81 (0.60, 1.10)
Model 1	1.00 (Reference)	0.57 (0.39, 0.81) **	0.73 (0.54, 1.00) *
Model 2	1.00 (Reference)	0.55 (0.38, 0.79) **	0.74 (0.38, 1.43)
IPTW Model	1.00 (Reference)	0.63 (0.44, 0.90) *	0.81 (0.60, 1.10)
Low dietary diversity			
Incidence (no. of deaths/1000 person-years)	4.99	4.05	4.73
Unadjusted Model	1.00 (Reference)	0.78 (0.46, 1.33)	0.92 (0.49, 1.73)
Model 1	1.00 (Reference)	0.76 (0.44, 1.29)	0.67 (0.36, 1.25)
Model 2	1.00 (Reference)	0.76 (0.45, 1.31)	0.66 (0.35, 1.24)
IPTW Model	1.00 (Reference)	0.78 (0.46, 1.33)	0.92 (0.49, 1.73)
High dietary diversity			
Incidence (no. of deaths/1000 person-years)	2.99	1.95	3.10
Unadjusted Model	1.00 (Reference)	0.65 (0.40, 1.07)	0.97 (0.67, 1.40)
Model 1	1.00 (Reference)	0.50 (0.30, 0.82) **	0.69 (0.47, 1.02)
Model 2	1.00 (Reference)	0.51 (0.31, 0.84) **	0.71 (0.48, 1.05)
IPTW Model	1.00 (Reference)	0.65 (0.40, 1.06)	0.96 (0.66, 1.39)
Low energy intake			
Incidence (no. of deaths/1000 person-years)	5.79	4.12	4.57
Unadjusted Model	1.00 (Reference)	0.78 (0.52, 1.17)	0.80 (0.56, 1.16)
Model 1	1.00 (Reference)	0.72 (0.47, 1.10)	0.83 (0.57, 1.21)
Model 2	1.00 (Reference)	0.75 (0.48, 1.14)	0.80 (0.53, 1.19)
IPTW Model	1.00 (Reference)	0.78 (0.52, 1.17)	0.80 (0.56, 1.17)
High energy intake			
Incidence (no. of deaths/1000 person-years)	3.20	1.06	2.16
Unadjusted Model	1.00 (Reference)	0.35 (0.16, 0.74) **	0.72 (0.43, 1.21)
Model 1	1.00 (Reference)	0.32 (0.15, 0.68) **	0.61 (0.36, 1.05)
Model 2	1.00 (Reference)	0.31 (0.14, 0.67) **	0.60 (0.35, 1.04)
IPTW Model	1.00 (Reference)	0.35 (0.16, 0.74) **	0.72 (0.43, 1.21)

*p < 0.05, ** *p* < 0.01. Model 1 adjusted: age, sex, education, individual annual income, place of residence. Model 2 based on model 1 further adjusted physical activity, smoking status, alcohol intake, vegetable intake, fruit intake, red meat intake, dietary diversity score, and energy intake. Dietary diversity score and energy intake were not adjusted in their corresponding stratified analyses. IPTW Model: Inverse probability of treatment weight cox proportion hazard regression to balance confounding factors among different groups of milk intake.

## Data Availability

Publicly available datasets were analyzed in this study. This data can be found here: https://www.cpc.unc.edu/projects/china (accessed on 6 January 2022).

## Supplementary Materials

The following supporting information can be downloaded at: https://www.mdpi.com/article/10.3390/nu14020292/s1, Table S1: Association between milk intake at baseline and all-cause mortality.
