# Supplementary material for: Association between Milk Intake and All-Cause Mortality among Chinese Adults: A Prospective Study
Source: Nutrients. 2022 Jan 11;14(2):292. doi: 10.3390/nu14020292 (PMC8779580; doi:10.3390/nu14020292)
Supplement: Supplementary file 1 [file nutrients-14-00292-s001.zip › nutrients-1531240-supplementary.pdf]

**Table S1.** Association between milk intake at baseline and all-cause mortality.

| | No consumption | 0.1-2 portions/week | >2 portions/week |
|---|---|---|---|
| **Overall population** | | | |
| Incidence (no.of deaths/1000 person-years) | 4.33 | 2.64 | 3.52 |
| Unadjusted Model | 1.00 (Reference) | 0.63 (0.47, 0.85) ** | 0.90 (0.67, 1.22) |
| Model 1 | 1.00 (Reference) | 0.60 (0.44, 0.81) ** | 0.82 (0.60, 1.11) |
| Model 2 | 1.00 (Reference) | 0.57 (0.42, 0.79) *** | 0.78 (0.57, 1.08) |
| IPTW Model | 1.00 (Reference) | 0.63 (0.47, 0.85) ** | 0.90 (0.67, 1.22) |
| **Low dietary diversity** | | | |
| Incidence (no.of deaths/1000 person-years) | 4.99 | 3.84 | 5.49 |
| Unadjusted Model | 1.00 (Reference) | 0.74 (0.46, 1.18) | 1.05 (0.59, 1.87) |
| Model 1 | 1.00 (Reference) | 0.72 (0.45, 1.16) | 0.73 (0.41, 1.31) |
| Model 2 | 1.00 (Reference) | 0.72 (0.44, 1.15) | 0.73 (0.41, 1.29) |
| IPTW Model | 1.00 (Reference) | 0.74 (0.46, 1.19) | 1.05 (0.60, 1.86) |
| **High dietary diversity** | | | |
| Incidence (no.of deaths/1000 person-years) | 3.00 | 2.21 | 3.12 |
| Unadjusted Model | 1.00 (Reference) | 0.71 (0.47, 1.06) | 1.04 (0.71, 1.52) |
| Model 1 | 1.00 (Reference) | 0.55 (0.36, 0.83) ** | 0.78 (0.53, 1.16) |
| Model 2 | 1.00 (Reference) | 0.57 (0.37, 0.86) ** | 0.79 (0.53, 1.18) |
| IPTW Model | 1.00 (Reference) | 0.70 (0.47, 1.06) | 1.04 (0.71, 1.52) |
| **Low energy intake** | | | |
| Incidence (no.of deaths/1000 person-years) | 5.85 | 4.21 | 4.51 |
| Unadjusted Model | 1.00 (Reference) | 0.75 (0.53, 1.06) | 0.85 (0.59, 1.24) |
| Model 1 | 1.00 (Reference) | 0.71 (0.50, 1.03) | 0.86 (0.59, 1.27) |
| Model 2 | 1.00 (Reference) | 0.73 (0.50, 1.07) | 0.80 (0.53, 1.20) |
| IPTW Model | 1.00 (Reference) | 0.75 (0.53, 1.06) | 0.85 (0.59, 1.24) |
| **High energy intake** | | | |
| Incidence (no.of deaths/1000 person-years) | 3.22 | 1.26 | 2.49 |
| Unadjusted Model | 1.00 (Reference) | 0.40 (0.22, 0.71) ** | 0.86 (0.52, 1.43) |
| Model 1 | 1.00 (Reference) | 0.40 (0.22, 0.72) ** | 0.78 (0.47, 1.31) |
| Model 2 | 1.00 (Reference) | 0.36 (0.19, 0.68) ** | 0.67 (0.39, 1.16) |
| IPTW Model | 1.00 (Reference) | 0.40 (0.22, 0.72) ** | 0.86 (0.52, 1.42) |

$^{**}$ $P < 0.05$, $^{***}$ $P < 0.01$.

Model 1 adjusted: age, gender, education, individual annual income, place of residence.

Model 2 based on model 1 further adjusted physical activity, smoke, alcohol, vegetables intake, fruits intake, and red meat intake, dietary diversity score, and energy intake. Dietary diversity score and energy intake were not adjusted in their corresponding stratified analyses.

IPTW Model: inverse probability of treatment weight (IPTW) cox proportion hazard regression to

balance confounding factors among different groups of milk intake.
